# Supplementary material for: Learning effect on an isokinetic knee strength test protocol among male adolescent athletes
Source: PLoS One. 2023 Jul 26;18(7):e0288382. doi: 10.1371/journal.pone.0288382 (PMC10370732; doi:10.1371/journal.pone.0288382)
Supplement: Attachment — Submitted filename: COMMENT 2 11052023 Learning effect on an isokinetic knee strength test protocol.pdf [file pone.0288382.s003.pdf]

COMMENT

Thank the authors for taking previous review advisees in preparing the presented study for publishing. Significant changes and improvements are noticeable. I will only suggest a few more corrections. The most important advice is to explain more about the differences between obtained results with the previous studies regarding both experimental questions. Father, to explain in more detail why the obtained mean difference and %CV between the first and second testing in KEcc and KEecc, is higher (is there a significant difference beside small ES)?

Overall, the study has provided exciting and valuable data regarding standardizing the protocol of isokinetic testing of youth athletes.

Some additional corrections and suggestions:

COMMENTS

Page 5, line 120: Can you please specify the range of ICC in previous studies, and type of motoric tasks or activity?

Page 6, line 159: There is no need to repeat the number of subjects.

Page 6-7, lines 159 - 164: This part should be in introduction. The number of the reference can be added to justify the sample size power.

Page 7, line 172: Can you please define how many days past between testing days (mean)?

Page 7, table 1: Suggestion - present as “mean±SD” instead “Mean Standard deviation”

Page 8, line 183: Without “described elsewhere” in sentence.

Page 8, line 184-185: A portable scale was used for measuring of body mass, please make the sentence clearer?
