# Supplementary material for: Learning effect on an isokinetic knee strength test protocol among male adolescent athletes
Source: PLoS One. 2023 Jul 26;18(7):e0288382. doi: 10.1371/journal.pone.0288382 (PMC10370732; doi:10.1371/journal.pone.0288382)
Supplement: Attachment — Submitted filename: 625_a_2_COMMENTS_point-per-point_20230228.docx [file pone.0288382.s002.docx]

Reviewers' comments:

Reviewer's Responses to Questions

**Comments to the Author**

1. Is the manuscript technically sound, and do the data support the conclusions?

The manuscript must describe a technically sound piece of scientific research with data that supports the conclusions. Experiments must have been conducted rigorously, with appropriate controls, replication, and sample sizes. The conclusions must be drawn appropriately based on the data presented.

Reviewer #1: Yes

Reviewer #2: Partly

2. Has the statistical analysis been performed appropriately and rigorously?

Reviewer #1: I Don't Know

Reviewer #2: Yes

3. Have the authors made all data underlying the findings in their manuscript fully available?

The [PLOS Data policy](https://emea01.safelinks.protection.outlook.com/?url=http%3A%2F%2Fwww.plosone.org%2Fstatic%2Fpolicies.action%23sharing&data=05%7C01%7C%7Ce74ed48eb2d144a4b4c908dae19dc30f%7C84df9e7fe9f640afb435aaaaaaaaaaaa%7C1%7C0%7C638070364949592835%7CUnknown%7CTWFpbGZsb3d8eyJWIjoiMC4wLjAwMDAiLCJQIjoiV2luMzIiLCJBTiI6Ik1haWwiLCJXVCI6Mn0%3D%7C3000%7C%7C%7C&sdata=bDV7yAFjzO0zfX%2Bh5ljetGG2HxULOqZW9iFiK7ILs8E%3D&reserved=0) requires authors to make all data underlying the findings described in their manuscript fully available without restriction, with rare exception (please refer to the Data Availability Statement in the manuscript PDF file). The data should be provided as part of the manuscript or its supporting information, or deposited to a public repository. For example, in addition to summary statistics, the data points behind means, medians and variance measures should be available. If there are restrictions on publicly sharing data—e.g. participant privacy or use of data from a third party—those must be specified.

Reviewer #1: Yes

Reviewer #2: Yes

4. Is the manuscript presented in an intelligible fashion and written in standard English?

PLOS ONE does not copyedit accepted manuscripts, so the language in submitted articles must be clear, correct, and unambiguous. Any typographical or grammatical errors should be corrected at revision, so please note any specific errors here.

Reviewer #1: No

Reviewer #2: No

5. Review Comments to the Author

Please use the space provided to explain your answers to the questions above. You may also include additional comments for the author, including concerns about dual publication, research ethics, or publication ethics. (Please upload your review as an attachment if it exceeds 20,000 characters)

**REVIEWER #1:**

Congratulations on a well organized study. Overall, I found the manuscript very interesting, and the study adds new information to this field. However, I would recommend obtaining help from an **English-speaking colleague to review the text** and make changes throughout (i.e. meanwhile instead of meantime) More specifically, here are further directed comments:

**AUTHORS:**

The revised version was edited by English-speaking colleague. Consequently, one author was added.

**REVIEWER #1:**

Introduction: did not convey "why" it is important to test strength in this age group, or why this study is needed right now.

**AUTHORS:**

The revised version now emphasizes the importance of data quality particularly in longitudinal studies and is citing two studies of young athletes adopting different number of repetitions to retain the best torque for analysis.

**REVIEWER #1:**

lines 79-86: can combine as a range of examples in the literature, vs citing each individually. It takes too long to get to the point.

**AUTHORS:**

The revised version was abbreviated and does not include information about warm-up and details of the sample in the cited studies.

**REVIEWER #1:**

lines 94-95: need more evidence to substanciate this affirmation

**AUTHORS:**

The revised version now includes an emphasis on the need to obtain reliable measurements in the context of youth sports.

Methods:

**REVIEWER #1:**

line 113: describe the training of the observer (have they done this many times?)
Information added. The tests were performed by a single trained observer who accumulated more than 500 assessments.

**AUTHORS:**

The information about the observer experience is now detailed.

**REVIEWER #1:**

line 116: reconsider the format to express age, seems odd with two decimal points currently

**AUTHORS:**

Adjusted

**REVIEWER #1:**

lines 143-144: not familiar if this is the standard method to correct for gravity with this device. please add reference.

**AUTHORS:**

Reference added.

**REVIEWER #1:**

line 156: describe what would be considered an "atypical curve". Were there data removed in the final analysis?

Information added

**AUTHORS:**

The sentence was adjusted, thanks.

**REVIEWER #1:**

lines 160-161: is the sample sufficient to power a multi-level regression analysis? If not, reconsider inclusion.

**AUTHORS:**

Sample size included is justified in the respective section. Level has 190 measurements, level 2 has 38 cases.

**REVIEWER #1:**

lines 168-171: if it was the intention to do a test-retest measurement, this should appear as a secondary goal at the end of the introduction

**AUTHORS:**

The aim was adjusted following your suggestion, thanks

**REVIEWER #1:**

line 171: "d-value"? Does this mean Cohen's d value? please specify

**AUTHORS:**

Added

Results:

**REVIEWER #1:**

line 189: wording needs to be softer, i.e. regression results "suggest" these are important factors

**AUTHORS:**

Authors agree. Corrected

**REVIEWER #1:**

Table 3: add missing abbreviation explanations in the table notes

**AUTHORS:**

Missing abbreviations were introduced

**REVIEWER #1:**

line 255: please reword so that you state that this is a hypothesis or a speculation

**AUTHORS:**

Changed. Hopefully, it is now more understandable

Discussion:

**REVIEWER #1:**

lines 273-274: please reword. Lacks clarity.

**AUTHORS:**

Clarified. Thanks.

**REVIEWER #1:**

line 300-301: the comparison with your findings should appear much earlier in this paragraph. Needs rewriting.

**AUTHORS:**

Modified accordingly

**REVIEWER #1:**

lines 311-312: please include description of this subsample -> do they represent the original sample well?

**AUTHORS:**

Mean values for chronological age, training experience, stature and body mass were now presented. They approached mean values of the total sample presented in Table 1

**REVIEWER #1:**

General:
Would you consider it relevant to compare relative strength results standardized by weight as well? The differences between repetitions may become more trivial statistically, but your main findings showing that an important group of participants only exhibit their maximal capacities in the last repetitions would remain true.

**AUTHORS:**

The conclusions were refined. The interesting note addressed by reviewer 1 would probably requires an allometric approach to the problem.

**Reviewer #2:**

COMMENT
The subject and results of presented study are of practical impotency for standardization of isokinetic dynamometer testing procedures. Invested effort for conducting and writing the paper is noticeable. However, some methodological corrections must be applied, as well as language improvements that are following almost the whole text, that should be revised and changed. I will address some of them with the following comments. It is important to notice that results are not fully presented in conjunction with tables and figures. The authors should include more information and use more clear language to present obtained results.

**AUTHORS:**

The manuscript in its revised version was improved according to comments addressed by both reviewers and English was revised by David Thivel.

**Reviewer #2:**

COMMENT

Page 4, lines (97 – 101): From methodologic point of view the hypotheses are missing, which is leading to later methodological problems.

**AUTHORS:**

Hypotheses were added at the end of the section introduction.

**Reviewer #2:**

COMMENT
Page 4, line 108: Specify participants characteristics in this section. Age of participance should be standardized through whole paper, I suggest as mean and standard deviation.

**AUTHORS:**

The range, mean and standard deviation was now included in the revised version.

**Reviewer #2:**

COMMENT
Page 5, line 115: Sample should go before procedures?

**AUTHORS:**

Thanks for the question. Authors do not agree and in all past manuscripts (including in BMC Pediatrics) the organization of the experiment including approval in ethics committee, distribution of informed consent precedes the sample. Let us wait for the editor-in-chief recommendations. If needed, authors will modify the structure of the methods section

**Reviewer #2:**

COMMENT
Page 5, line 125: The name of reference is missing in a text form?

**AUTHORS:**

Corrected. Hopefully, it is now understandable for readers.

**Reviewer #2:**

COMMENT
Page 5, line 131: This should be subchapter Experimental procedures?

Anthropometry and isokinetic dynamometer are present now as subchapters of procedures.

**AUTHORS:**

Let us wait for the editor-in-chief recommendations. If needed, authors will modify the structure of the methods section

**Reviewer #2:**

COMMENT
Page 7, line 168: Subsample of 17 swimmers is not mentioned in sample subchapter, what are the specific characteristic of this sample, are there differences between this subgroup and mane sample?

**AUTHORS:**

Now added. Thanks.

**Reviewer #2:**

Also in this case you have not precisely formulated the aim and hypothesize for determination of reliability of executed isokinetic test…

**AUTHORS:**

The final sentence of the last paragraph in the introduction was revised accordingly to this comment. Thanks.

**Reviewer #2:**

COMMENT
Page 7, line 171: Please specify what effect size measure have you used, Cohen’s d or some other?
It was added.

**AUTHORS:**

**Reviewer #2:**

COMMENT
Page 7, line 173: The Bland-Altman plot analysis is evaluating a bias between the mean differences, and to in your case estimate an agreement interval between repeated measurements. Suggestion is to correct differences into agreements.

**AUTHORS:**

The term “differences” was replaced by “agreements” as suggested.

**Reviewer #2:**

COMMENT
Page 8, line (185): Table 1 should be separated into two tables, one with Descriptive morphological data (on chronological age, training experience, body size), and second one with isokinetic strength outputs. Also, Table 1 should have characteristics of subsample, or it can be in separated table.

**AUTHORS:**

Modified as suggested.

**Reviewer #2:**

COMMENT
Page 8, line 183: No need to repeat subjects characteristics.

**AUTHORS:**

Characteristics of the participants were now concentrated in Table 1 and are not repeated in the text.

**Reviewer #2:**

COMMENT
Page 8, lines (220-228): It is very difficult the follow the description of results in correlation with figures and tables.

**AUTHORS:**

Hopefully the revised version is now easier to follow.

**Reviewer #2:**

Lines 220 – 224: The text should in subchapter that describes sample and procedures.

**AUTHORS:**

Done

**Reviewer #2:**

Lines 227 – 228: What exactly several participants mean, how many of them exactly. This is not mentioned in discussion?

**AUTHORS:**

Detailed information about the number of cases who decreased their highest PT was introduced.

**Reviewer #2:**

Page 11, lines (243-260): The authors should clarify the following section in correlation with presented hypotheses, aims, and results, to avoid confusion. The language should be revised to improve the readability.

**AUTHORS:**

Improved

**Reviewer #2:**

Line 246: This is not in correlation with previous mentioned sample age.

**AUTHORS:**

Corrected

**Reviewer #2:**

COMMENT
Page 13, lines (299-300): There is no need to repeat the protocol.

**AUTHORS:**

Thanks. Repetition of the protocol was deleted.

**Reviewer #2:**

COMMENT
Pages 3-12: Reference should be added to the end of sentences in next lines: 74, 75, 78, 92, 94, 96, 274.

**AUTHORS:**

Added accordingly to recommendations

**Reviewer #2:**

COMMENT
Pages 3-12: Better sentence formulation: lines 75 – 78, 86 – 90, 95 – 96, 97 – 101, 109 – 112, 161 – 164.

**AUTHORS:**

Refined by Professor David Thivel who was introduced as co-author. Hopefully, the manuscript is now understandable.
