# Supplementary material for: Learning effect on an isokinetic knee strength test protocol among male adolescent athletes
Source: PLoS One. 2023 Jul 26;18(7):e0288382. doi: 10.1371/journal.pone.0288382 (PMC10370732; doi:10.1371/journal.pone.0288382)
Supplement: Attachment — Submitted filename: 658_a_2_Response to reviewers_MJCS_JVS_DVM_20230614.docx [file pone.0288382.s004.docx]

PONE-D-22-24405R1

Learning effect on an isokinetic knee strength test protocol among male adolescent athletes

Dear Dr. Coelho-e-Silva,

Thank you for submitting your manuscript to PLOS ONE. After careful consideration, we feel that it has merit but does not fully meet PLOS ONE’s publication criteria as it currently stands. Therefore, we invite you to submit a revised version of the manuscript that addresses the points raised during the review process.

=============================================================

ACADEMIC EDITOR:

Dear Authors,

two experts in the field reviewed your revised manuscript version founding some minor points you should consider in the revision process.

Please submit your revised manuscript by Jun 29 2023 11:59PM. If you will need more time than this to complete your revisions, please reply to this message or contact the journal office at plosone@plos.org. When you're ready to submit your revision, log on to https://www.editorialmanager.com/pone/ and select the 'Submissions Needing Revision' folder to locate your manuscript file.

Please include the following items when submitting your revised manuscript:

A rebuttal letter that responds to each point raised by the academic editor and reviewer(s). You should upload this letter as a separate file labeled 'Response to Reviewers'.

A marked-up copy of your manuscript that highlights changes made to the original version. You should upload this as a separate file labeled 'Revised Manuscript with Track Changes'.

An unmarked version of your revised paper without tracked changes. You should upload this as a separate file labeled 'Manuscript'.

If you would like to make changes to your financial disclosure, please include your updated statement in your cover letter. Guidelines for resubmitting your figure files are available below the reviewer comments at the end of this letter.

If applicable, we recommend that you deposit your laboratory protocols in protocols.io to enhance the reproducibility of your results. Protocols.io assigns your protocol its own identifier (DOI) so that it can be cited independently in the future. For instructions see: https://journals.plos.org/plosone/s/submission-guidelines#loc-laboratory-protocols. Additionally, PLOS ONE offers an option for publishing peer-reviewed Lab Protocol articles, which describe protocols hosted on protocols.io. Read more information on sharing protocols at https://plos.org/protocols?utm_medium=editorial-email&utm_source=authorletters&utm_campaign=protocols.

We look forward to receiving your revised manuscript.

Kind regards,

Emiliano Cè

Academic Editor

PLOS ONE

Journal Requirements:

Please review your reference list to ensure that it is complete and correct. If you have cited papers that have been retracted, please include the rationale for doing so in the manuscript text, or remove these references and replace them with relevant current references. Any changes to the reference list should be mentioned in the rebuttal letter that accompanies your revised manuscript. If you need to cite a retracted article, indicate the article’s retracted status in the References list and also include a citation and full reference for the retraction notice.

AUTHORS:

The revised version was carefully checked.

=============================================================

Reviewer's Responses to Questions

Comments to the Author

1. If the authors have adequately addressed your comments raised in a previous round of review and you feel that this manuscript is now acceptable for publication, you may indicate that here to bypass the “Comments to the Author” section, enter your conflict of interest statement in the “Confidential to Editor” section, and submit your "Accept" recommendation.

Reviewer #1: (No Response)

Reviewer #2: All comments have been addressed

2. Is the manuscript technically sound, and do the data support the conclusions?

The manuscript must describe a technically sound piece of scientific research with data that supports the conclusions. Experiments must have been conducted rigorously, with appropriate controls, replication, and sample sizes. The conclusions must be drawn appropriately based on the data presented.

Reviewer #1: Partly

Reviewer #2: Yes

3. Has the statistical analysis been performed appropriately and rigorously?

Reviewer #1: Yes

Reviewer #2: Yes

4. Have the authors made all data underlying the findings in their manuscript fully available?

The PLOS Data policy requires authors to make all data underlying the findings described in their manuscript fully available without restriction, with rare exception (please refer to the Data Availability Statement in the manuscript PDF file). The data should be provided as part of the manuscript or its supporting information, or deposited to a public repository. For example, in addition to summary statistics, the data points behind means, medians and variance measures should be available. If there are restrictions on publicly sharing data—e.g. participant privacy or use of data from a third party—those must be specified.

Reviewer #1: Yes

Reviewer #2: Yes

5. Is the manuscript presented in an intelligible fashion and written in standard English?

PLOS ONE does not copyedit accepted manuscripts, so the language in submitted articles must be clear, correct, and unambiguous. Any typographical or grammatical errors should be corrected at revision, so please note any specific errors here.

Reviewer #1: No

Reviewer #2: Yes

6. Review Comments to the Author

Please use the space provided to explain your answers to the questions above. You may also include additional comments for the author, including concerns about dual publication, research ethics, or publication ethics. (Please upload your review as an attachment if it exceeds 20,000 characters)

Reviewer #1: Thank you for editing this new version of your manuscript with careful consideration for the comments from both reviewers. This version is a very significant improvement for international readers.

**AUTHORS:**

Thanks.

I would recommend further attention to the following comments for the next round:

In general, try to decrease the length of your sentences to avoid syntax errors and to make reading easier to follow for your audience.

**AUTHORS:**

Authors have reduced the sentences and the manuscript was edited by David Thivel who obtained the PhD in Canada.

- Objective 2: state as test-retest reliability for clarity. Current wording makes it harder to distinguish from first objective.

**AUTHORS:**

Refined as suggested.

- line 78: "meantime" should be "meanwhile" (as per round 1 comments)

**AUTHORS:**

Changed as suggested.

- although improved, paragraph 1 of the introduction still for me does not convey the importance of your main point: protocols differ between studies, sometimes 3 or 5 (or more). Validity of shorter protocols may be compromised if the peak torque is not obtained in the first three attempts.

**AUTHORS:**

Thanks for the comment. The essential is now in the final sentence of the abstract and adequately commented across the manuscript. The revised version is now more objective and concise.

- lines 95 to 103: needs rewriting. The content is there, but syntax is not at the correct level

**AUTHORS:**

Adjusted as recommended.

- line 111: avoid using reference [15] about multiple sclerosis. This population has specific fatigue problems related to their neurological condition that cannot be compared with healthy youth swimmers

**AUTHORS:**

Deleted.

- lines 119/120: sentence appears incomplete. add what the ICC values are?

**AUTHORS:**

Adjusted as recommended.

- lines 130/131: as previously mentioned, use term test-retest reliability for the international audience

**AUTHORS:**

Changed as recommended across the entire manuscript.

- lines 155/159: the sample size calculation here does not work. You are using the number of measurements instead of the number of participants. The sentence described is coherent to evaluate the sample needed to power the test-retest comparison. You also need to state what the expected difference (effect size) would be between groups to justify the sample size.

**AUTHORS:**

Information regarding calculation of power sample is now included for the various analysis.

- lines 159-164: good information, but should be moved to either the introduction or the discussion

**AUTHORS:**

Done as suggested.

- line 183: use the term "height" instead of stature for international audience. Change in Table 1 as well

**AUTHORS:**

Stature refers to the specific height of the vertex. In all previous studies, authors adopt the term stature.

- line 196: "strips" should be the word "straps"

**AUTHORS:**

Thanks. Changed.

- line 210: how was the data filtered?

- lines 214-215: this seems backwards. Would normally expect the raw data to be filtered to remove noise, then corrected for limb inertia, and then select only the data from the target speed (+/- 5%). Described as such, it seems you followed a different process.

**AUTHORS:**

The revised version now includes the information.

- line 229: define what you mean by "technical error of measurement"

**AUTHORS:**

Detailed.

- lines 250-252: leave interpretation sentences for the discussion, does not belong in the Results

**AUTHORS:**

Agree. Deleted.

- Table 1: much improved, good to see the information for both groups here

**AUTHORS:**

Thanks.

- Table 2: must explain why there is not consistently the same number of participants for each rep? Is this related to the data processing question from lines 214-215? How do you explain that the ICC for KFcc is lower despite the values being more similar across the 5 repetitions?

**AUTHORS:**

The following was added in Table 2: * For each repetition, when PT was not obtained within 95% of the settled angular velocity, data was not retained for analysis.

Multilevel structures do not require balanced data to obtain eﬃcient estimates. In other words, it is not necessary to have the same number of lower-level units within each higher-level unit. With repeated measures data we do not require the same number of measurement occasions per individual subject (level 2).

In this type of model (i.e., variance components model), the residual variance is partitioned into components corresponding to each 2 level in the hierarchy and the variance between repetition and the variance between individuals within a given repetition are obtained. ICC, provides the proportion of the total residual variation.

Briefly, the apparently stable mean values for each repetitions combined to lower ICC for KFcc, indicate large intra-individual fluctuation of performance scores and inter-individual variance. The PT value is not fully stable across the five repetitions.

- lines 301/309: paragraph is not clear enough, needs to be rewritten

**AUTHORS:**

Improved, substantially.

- lines 311/313: link this information with your findings. it seems lost otherwise

**AUTHORS:**

linked.

- line 339: i believe you are presenting your values, please make it explicit for the reader

**AUTHORS:**

It is now explicit.

- lines 372/374: I appreciate that you considered my comment about relative strength measurements, but I would like you to confirm that you have proceeded with this calculation and confirmed that your statistical conclusions remain the same

**AUTHORS:**

Figures were revised and now refers to error in N.m per unit of body mass, that is, N.m/kg

Reviewer #2: COMMENT

Thank the authors for taking previous review advisees in preparing the presented study for publishing. Significant changes and improvements are noticeable. I will only suggest a few more corrections. The most important advice is to explain more about the differences between obtained results with the previous studies regarding both experimental questions. Father, to explain in more detail why the obtained mean difference and %CV between the first and second testing in KEcc and KEecc, is higher (is there a significant difference beside small ES)?

Overall, the study has provided exciting and valuable data regarding standardizing the protocol of isokinetic testing of youth athletes.

**AUTHORS:**

Thanks. In fact, Table 2 confirms that the magnitude of changes in mean values between testing sessions is negligible (small). This is well illustrated in Figures. However, %CV is larger for knee extensors than for knee flexors. Figure 2 has now identical Y-axis for KE and KF. This is now adequately commented in the main findings at discussion section.

Some additional corrections and suggestions:

COMMENTS

Page 5, line 120: Can you please specify the range of ICC in previous studies, and type of motoric tasks or activity?

**AUTHORS:**

The range of ICCs and type of activities were added to the introduction. Thanks.

Page 6, line 159: There is no need to repeat the number of subjects.

**AUTHORS:**

Adjusted.

Page 6-7, lines 159 - 164: This part should be in introduction. The number of the reference can be added to justify the sample size power.

**AUTHORS:**

The sentence was moved to the introduction. The following reference was added to justify the sample size used on the paired-t test analysis:

van Tittelboom, Vanessa et al. “Reliability of Isokinetic Strength Assessments of Knee and Hip Using the Biodex System 4 Dynamometer and Associations With Functional Strength in Healthy Children.” Frontiers in sports and active living vol. 4 817216. 24 Feb. 2022, doi:10.3389/fspor.2022.817216

Page 7, line 172: Can you please define how many days past between testing days (mean)?

**AUTHORS:**

This point is now clarified.

Page 7, table 1: Suggestion - present as “mean±SD” instead “Mean Standard deviation”

**AUTHORS:**

Adjusted.

Page 8, line 183: Without “described elsewhere” in sentence.

**AUTHORS:**

Adjusted.

Page 8, line 184-185: A portable scale was used for measuring of body mass, please make the sentence clearer?

**AUTHORS:**

The sentence was adjusted.

7. PLOS authors have the option to publish the peer review history of their article (what does this mean?). If published, this will include your full peer review and any attached files.

If you choose “no”, your identity will remain anonymous but your review may still be made public.

Do you want your identity to be public for this peer review? For information about this choice, including consent withdrawal, please see our Privacy Policy.

Reviewer #1: Yes: Félix Croteau

Reviewer #2: No

[NOTE: If reviewer comments were submitted as an attachment file, they will be attached to this email and accessible via the submission site. Please log into your account, locate the manuscript record, and check for the action link "View Attachments". If this link does not appear, there are no attachment files.]

While revising your submission, please upload your figure files to the Preflight Analysis and Conversion Engine (PACE) digital diagnostic tool, https://pacev2.apexcovantage.com/. PACE helps ensure that figures meet PLOS requirements. To use PACE, you must first register as a user. Registration is free. Then, login and navigate to the UPLOAD tab, where you will find detailed instructions on how to use the tool. If you encounter any issues or have any questions when using PACE, please email PLOS at figures@plos.org. Please note that Supporting Information files do not need this step.

In compliance with data protection regulations, you may request that we remove your personal registration details at any time. (Remove my information/details). Please contact the publication office if you have any questions.
