# Supplementary material for: Learning effect on an isokinetic knee strength test protocol among male adolescent athletes
Source: PLoS One. 2023 Jul 26;18(7):e0288382. doi: 10.1371/journal.pone.0288382 (PMC10370732; doi:10.1371/journal.pone.0288382)
Supplement: Attachment — Submitted filename: COMMENT 3 126062023 Learning effect on an isokinetic knee strength test protocol.doc [file pone.0288382.s005.doc]

COMMENT

Thank the authors for taking previous review advisees in preparing the presented study for publishing. The study has provided exciting and valuable data regarding standardizing the protocol of isokinetic testing of youth athletes.

I wish a lot of success in a future research work.
